# Specific Chemical and Genetic Markers Revealed a Thousands-Year Presence of Toxic *Nodularia spumigena* in the Baltic Sea

**DOI:** 10.3390/md16040116

**Published:** 2018-04-04

**Authors:** Marta Cegłowska, Anna Toruńska-Sitarz, Grażyna Kowalewska, Hanna Mazur-Marzec

**Affiliations:** 1 Institute of Oceanology, Polish Academy of Sciences, Powstańców Warszawy 55, PL-81-727 Sopot, Poland; mceglowska@iopan.pl (M.C.); kowalewska@iopan.gda.pl (G.K.); 2 Division of Marine Biotechnology, Faculty of Oceanography and Geography, University of Gdańsk, Marszałka J. Płisudskiego 46, PL-81-378 Gdynia, Poland; anna.torunska@ug.edu.pl

**Keywords:** nodularin, toxin, anabaenopeptins, molecular markers, cyanobacteria, Baltic Sea

## Abstract

In the Baltic Sea, diazotrophic cyanobacteria have been present for thousands of years, over the whole brackish water phase of the ecosystem. However, our knowledge about the species composition of the cyanobacterial community is limited to the last several decades. In the current study, the presence of species-specific chemical and genetic markers in deep sediments were analyzed to increase the existing knowledge on the history of toxic *Nodularia spumigena* blooms in the Baltic Sea. As chemical markers, three cyclic nonribosomal peptides were applied: the hepatotoxic nodularin, which in the sea was detected solely in *N. spumigena*, and two anabaenopeptins (AP827 and AP883a) characteristic of two different chemotypes of this species. From the same sediment samples, DNA was isolated and the gene involved in biosynthesis of nodularin, as well as the phycocyanin intergenic spacer region (PC-IGS), were amplified. The results of chemical and genetic analyses proved for the first time the thousands-year presence of toxic *N. spumigena* in the Baltic Sea. They also indicated that through all this time, the same two sub-populations of the species co-existed.

## 1. Introduction

Around 8500 BP, the intrusion of saline water from Kattegat to the Baltic led to the development of halocline and poor oxygenation of bottom layers. Consequently, the release of phosphorous from sediments created favorable conditions for the growth of N_2_-fixing cyanobacteria. The history of cyanobacteria in the Baltic Sea was previously reconstructed based on the analysis of chemical markers such as zeaxanthin, echinenone, δ^15^N isotope, and molybdenum [1,2,3]. However, these biomolecules are not specific enough to determine the species composition of phytoplankton community; they only enable the classification of organism to a general taxonomic group, like phylum [4]. In the 19th century, the macroscopic and microscopic analyses of field samples allowed for the first time to document the presence of *Nodularia spumigena* in the Baltic Sea [5]. In the following decades, occurrence of this cyanobacterium was rather rare [5]. Since the 1970s, as a consequence of human-induced eutrophication, the mass development of *N. spumigena* has become a regular phenomenon [5,6]. In the late 1980s, the production of nodularin (NOD) by the Baltic *N. spumigena* was reported for the first time [7]. This hepatotoxic cyclic peptide belongs to potent inhibitors of eukaryotic protein phosphatases and acts as tumor initiator and promoter in liver cells [8,9]. Nodularin is considered to be the most abundant natural toxin in the Baltic Sea [10]. In cyanobacterial bloom samples and in cultured *N. spumigena* isolates, a vast array of other bioactive metabolites with unknown effects on humans and aquatic biota was also identified [11,12,13,14]. 

To broaden our knowledge about the occurrence of toxic *N. spumigena* in the earlier stages of the Baltic Sea, NOD and the gene *mcyE/ndaF* involved in the biosynthesis of microcystin/nodularin were used as highly specific markers. These biomolecules were analyzed in long (LC) and short (SC) sediment cores collected from Gdansk Deep (Southern Baltic). Although nodularin has occasionally been detected in other cyanobacterial species [15,16,17,18], in the sea the toxin was solely identified in *N. spumigena*. Therefore, we assumed that the documented presence of NOD or the genes in the analyzed sediment layers would unequivocally indicate the occurrence of this species. To support our findings and to get more insight into the diversity of the Baltic *N. spumigena*, we additionally analyzed other nonribosomal peptides as well as the phycocyanin intergenic spacer (IGS) region between phycocyanin subunit genes *cpcB* and *cpcA* and its flanking regions (*cpcBA*-IGS). The collected data unequivocally and for the first time proved the thousands-year presence of the toxic *N. spumigena* in the Baltic Sea.

## 2. Results

### 2.1. Extraction and LC-MS/MS Analysis of NOD

Recovery of NOD from different sediment layers was similar and amounted to 91.6 ± 2.9% (2–4 cm SC), 96.3 ± 5.8% (64–66 cm LC), and 96.7 ± 6.4% (166–168 cm LC). In the analyzed samples, the peptide was identified based on its retention time (9.54 min), the proportions of three multiple reaction monitoring (MRM) transitions (two qualifier ions and one quantifier ion) (Supplementary Material Figure S1A,B), and the fragmentation spectrum (Supplementary Material Figure S1C,D). None of the applied methods indicated the presence of microcystins (MCs), the cyclic hepatotoxic peptides mainly produced by freshwater cyanobacteria. In all sediment samples, including the deepest layer (380–382 cm), the concentration of NOD was above the limit of MS/MS detection (LD = 0.05 ng/g dw).

**Figure 1:**
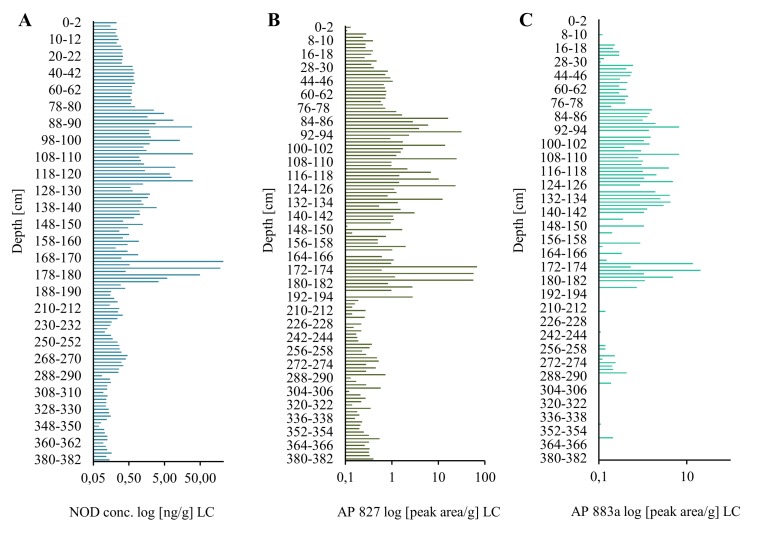
Nodularin (NOD) content (**A**) and relative amounts of anabaenopeptin AP827 (**B**) and AP883a (**C**) (peak area/g) in the long sediment core (LC) collected in the Gulf of Gdańsk, Southern Baltic.

**Figure 2:**
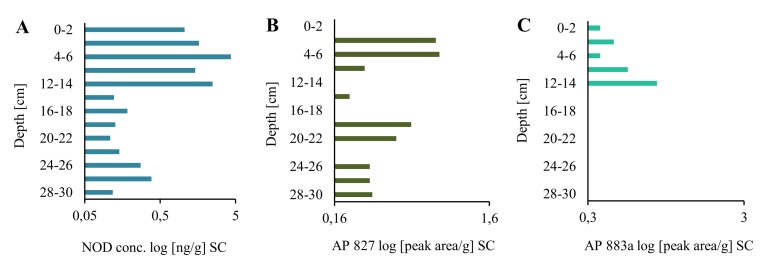
Nodularin (NOD) content (**A**) and relative amounts of anabaenopeptin AP827 (**B**) and A883a (**C**) [peak area/g] in the short sediment core (SC) collected in the Gulf of Gdańsk, Southern Baltic.

In the long core, concentrations of the peptide in lyophilized samples of sediments (dw) changed in an irregular way (Figure 1A; Supplementary Material Table S1). However, some trends in the changes can be observed. In the deepest 2-m part of the core (184–382 cm), concentrations of NOD were low and varied within a narrow range of 0.10–0.46 ng/g (Figure 1A; Supplementary Material Table S1). Significantly higher NOD concentrations, with the peak value of 225.94 ng NOD/g were detected in 170–176-cm layers. After a sharp drop (168–170 cm; 0.31 ng NOD/g), an increase in the concentration of the peptide up to 29.98–31.44 ng NOD/g was noted in layers 90–120 cm. From layer 78–80 cm, NOD concentrations decreased to 0.15–0.26 ng NOD/g in the top layers of the LC. Generally, the concentration of NOD in the uppermost part of LC was similar to NOD concentration in deeper parts (14–30 cm) of the short core (Figure 2A). In the most recent sediments (0–14 cm SC), at least one order of magnitude higher concentrations of NOD (1.05–4.32 ng NOD/g) were recorded (Figure 2A).

Apart from NOD, in many sediment samples from both cores, two anabaenopeptins (APs) were detected (Figure 1B,C and Figure 2B,C; Supplementary Material Table S1). Based on the fragmentation spectra, their structures were elucidated as AP827 Phe+CO[Lys+Val+Hty+MeAla+Phe] and AP883a Ile+CO[Lys+Met+Hph+MeHph+Met] (Supplementary Material Figures S2 and S3). The sediment layers with the highest intensities of AP ions in MRM chromatograms were also characterized by peak concentrations of NOD. The calculated Spearman correlations among the three cyanopeptides were positive (r_s_ = 0.73 for NOD/AP827, r_s_ = 0.63 for NOD/AP883a, and r_s_ = 0.79 for AP827/AP883a; *p* < 0.05). In majority of the samples, the signal intensities of MRM transitions for AP827 (*m*/*z* 828) were higher than for anabaenopeptin AP883a (*m*/*z* 884). In sediment layers with the lowest concentration of NOD, anabaenopeptin AP883a was not found (Figure 1A,C and Figure 2A,C).

### 2.2. Genetic Analysis of Sediment Samples

For genetic analysis, 23 sediment samples from various parts of the core containing different amounts of NOD were selected. This included: 20 sediment layers (2 cm) of the long core and two layers of the short core. In addition, the integrated sample containing homogenized material from 2–6 cm layers of the SC was used (Supplementary Material Table S2). FastDNA™ kit was most effective in DNA extraction from both SC and LC sediment samples. From two layers of SC, 266 ng DNA/µL (0–2 cm) and 202 ng DNA/µL (4–6 cm) were obtained. With the depth, the quantities of the extracted DNA decreased, and in layers deeper than 178 cm they were below 40 ng/µL. NucleoSpin^®^ Soil kit, buffer SL2 gave better results for SC sediments, while buffer SL1 was more effective in DNA extraction from all LC sediment samples (Supplementary Material Table S3). Regardless of the applied DNA extraction reagents, the clean-up procedure with the Anty-Inhibitor kit resulted in lower amounts of DNA and its quality expressed by A_260/280_ coefficient was not significantly improved (Supplementary Material Table S3). Although most of the extracted sedimentary DNA was of low quality, the amplification of PC-IGS gave PCR products (approx. 700 bp) for all analyzed samples and the amplification of *mcyE/ndaF* gene gave PCR products (approx. 500 bp) for nine out of 23 sediment samples (Supplementary Material Table S2). 

**Figure 3:**
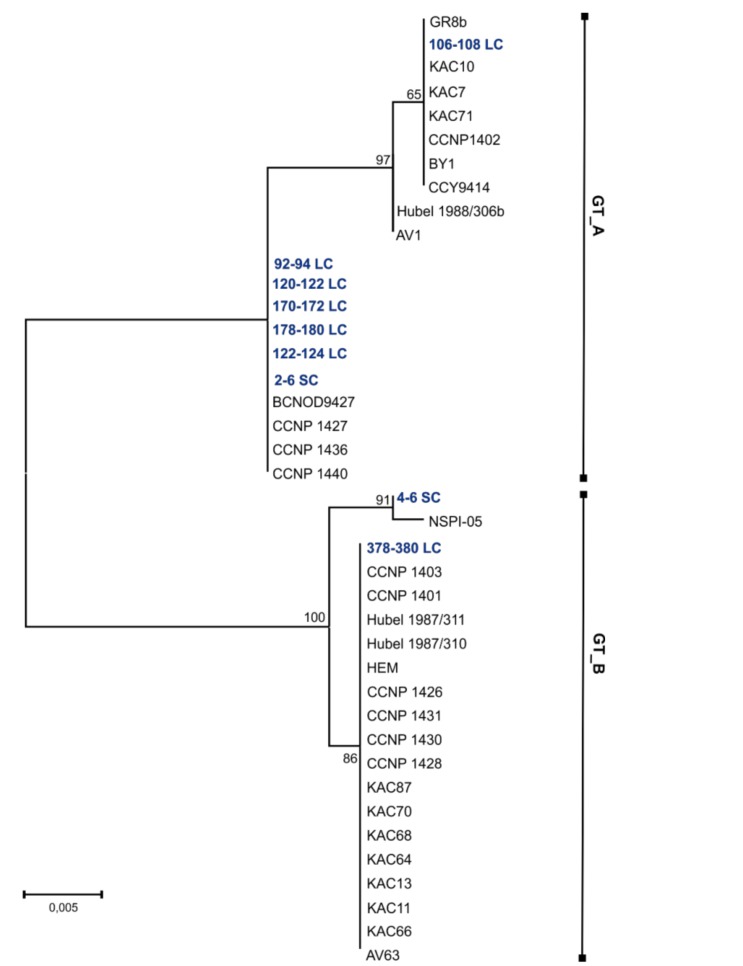
Maximum-likelihood (ML) phylogenetic tree based on the *cpcBA*-IGS sequences (496 bp) obtained from DNA isolated from Baltic sediments (marked in blue) and references *cpcBA*-IGS sequences (retrieved from NCBI) from *N. spumigena* strains (marked in black). Phylogenetic relationships were bootstrapped 1000 times. The branches with less than 50% bootstrap are shown as unresolved. Similar *cpcBA*-IGS sequences are marked as GT_A and GT_B, respectively (GT–genotype).

Good quality PC-IGS sequences (~500 bp) were obtained for nine of the 23 selected sediment samples (Supplementary Material Table S2). The PC-IGS regions from sediment samples showed 99–100% similarity to the PC-IGS regions from their nearest *N. spumigena* phylogenetic neighbors (sequences retrieved from GenBank). In the phylogenetic analysis, nine sedimentary PC-IGS sequences and thirty one PC-IGS sequences from the Baltic strains of *N. spumigena* (retrieved from GenBank) were included. The topologies of the constructed maximum-likelihood (ML), neighbor-joining (NJ), and maximum parsimony (MP) trees were similar (Figure 3; Supplementary Material Figure S4). Based on the occurrence in the tree, the PC-IGS from sediments were classified to one of the two clusters: GT_A or GT_B. In the genetic cluster GT_A, six PC-IGS sequences from LC (layers 92–94, 106–108, 120–122, 122–124, 170–172, and 178–180 cm) and one sequence from SC (2–6 cm) grouped together with the sequences from twelve *N. spumigena* strains. The remaining two sedimentary PC-IGS sequences (SC 4–6 cm and LC 378–380 cm) fell in one group with eighteen *N. spumigena* strains representing genotype GT_B (Figure 3). Pairwise distances among sequences from sediments and strains from the same genotype varied between 0 and 0.01, while p-distances among sequences from distinct genotypes differed in the range 0.04–0.05.

## 3. Discussion

Analysis of nodularin, anabaenopeptins, and PC-IGS sequences in deep sediments revealed a long-lasting presence of toxic *N. spumigena* in the Baltic Sea. Unfortunately, the sediment dating was not included in this work and precise time determination was not possible. However, the depth of the deepest sediment layers in which NOD was detected corresponds to the layers of the radiocarbon dated core collected previously from the same station (P116) and determined to be approximately 4000 years old [19]. This fact let us conclude that the toxic blooms of the cyanobacterium occurred in the Baltic Sea thousands of years ago, i.e., a long time before the human-induced eutrophication of this ecosystem.

The persistence of NOD in deep sediments can be attributed to high stability of the peptide. In the case of the structurally similar microcystins, a resistance to common proteases was observed [20]. As a consequence, only specific strains of bacteria were capable of microcystin degradation. Few of the strains were also able to degrade NOD [21]. Both cyanopeptides are relatively stable under different physico-chemical conditions, such as high range of salinity, extreme temperature, and pH [22,23,24,25].

The detection of NOD in all sediment samples indicates permanent occurrence of *N. spumigena* in the Baltic Sea. NOD is produced constitutively and environmental conditions have only minor effect on the rate of its biosynthesis [26]. In laboratory studies, more pronounced differences in cyanopeptide cell quota were observed only under extreme conditions. Therefore, we assumed that NOD concentration in sediments roughly corresponds with the changes in *N. spumigena* biomass generated in a given time period. The chemical evidence of long-lasting *N. spumigena* presence in the Baltic Sea was additionally supported by analysis of DNA molecular markers. Although the quantity and quality of the DNA isolated from sediments were generally low, we managed to obtain the PC-IGS and *mcyE*/*ndaF* PCR products from several sediment samples. However, despite our attempts to optimize the method, the sequencing of the *mcyE*/*ndaF* PCR product was not successful. The application of genetic markers in the analysis of old sediments still faces serious methodological constrains. A limited number of studies in which these markers were used as marine cyanobacteria tracers have been published [27,28,29,30,31,32,33,34,35,36,37,38,39,40,41,42]. In most of the studies, the presence of cyanobacteria was reconstructed based on 16S rRNA (Supplementary Material Table S4). As phycocyanin intergenic spacer is a highly variable region, it better reflects the diversity of cyanobacteria than 16S rRNA [13,43,44,45]. We succeeded in sequence determination of the sedimentary PC-IGS fragments. They were found to be closely related to the Baltic *N. spumigena* PC-IGS sequences deposited in GenBank. Close grouping of PC-IGS from different layers of the LC with the sequences from the Baltic *N. spumigena* isolates constitutes an additional and strong proof for a permanent presence of the species in the Baltic Sea. 

Our previous studies revealed a 27-year co-existence of two Baltic *N. spumigena* subpopulations (A and B) [13]. These subpopulations were distinguished based on the analyses of the produced anabaenopeptin variants and the sequences of PC-IGS region. The application of these molecular markers in the current work enabled us to gain better insight into the structure of *N. spumigena* population in earlier stages of the Baltic Sea. In many layers of the sediment core two anabaenopeptins, AP883a and AP827, were detected. Positive correlation of their concentration values in LC confirmed the same origin of the peptides. AP883a was previously found to be produced by all strains of the Baltic *N. spumigena* classified to subpopulation A, while AP827 was detected in isolates classified to subpopulation B [13]. The presence of the two subpopulations was also proved by analysis of DNA molecular markers. PC-IGS sequences obtained from different sediment layers were identical to PC-IGS sequences from the Baltic *N. spumigena* isolates representing either subpopulation A or subpopulation B. Therefore, the results of our analysis not only proved the long history of toxic *N. spumigena* blooms in the Baltic Sea, but also indicated that during this time the same two subpopulations representing two different chemotypes and genotypes of the species co-existed. The stability of the same cyanobacterial subpopulations in aquatic ecosystems was previously reported [13,46], but it has only been documented for less than the last three decades. So far, the selective biotic and abiotic forces driving the survival strategies and stabilizing the structure of cyanobacterial population have not been fully recognized [46,47,48]. 

Since the development of toxic *N. spumigena* requires higher summer temperature [49,50], the increase in the biomass of the cyanobacterium in warmer years could be expected. Therefore, we compared the tendency in the changes in NOD concentration with the existing knowledge about the European climate in the last 4000 years. Based on the results of ^14^C-dating of the core previously collected at the same station in the Gulf of Gdańsk (P116) [19], it may be assumed that the sediment layers with the increased NOD concentrations (90–180 cm) were deposited during Roman Climate Optimum. This period lasted several centuries, approximately from 2500 BP to 1600 BP, and was characterized by average summer temperature only slightly lower than in the late 20th century summers [51]. The explosive volcanic eruptions in 535–536 AD resulted in a cooling of North Hemisphere climate for several next decades [51,52]. At that time, environmental conditions were probably less favorable for *N. spumigena* growth. The increase in the content of NOD and APs in the uppermost part of the most recent sediments (SC) implies higher biomass of *N. spumigena* generated in the last decades. This tendency in NOD changes can be attributed to human-induced eutrophication and climate warming [53,54]. 

## 4. Materials and Methods

### 4.1. Sampling

Long sediment core (LC; 382 cm) was collected with a vibrocorer VKG-6/3 from station P116 located in the Gulf of Gdańsk (Gdańsk Deep; 54°39,04′ N, 19°17,24′ E) during a research cruise of r/v “IMOR” on 10 April 2015. From the same location, a short core (SC) was collected with Nemistö gravity corer and the upper 30-cm section of the core was analyzed. Both cores were sliced into 2-cm-thick layers. The subsamples intended for chemical analysis were lyophilized and stored in desiccator at room temperature. Sediment subsamples intended for genetic analysis were immediately frozen and stored at −80 °C.

### 4.2. Extraction of Cyanopeptides

To test the recovery of NOD from sediments, three different samples were analyzed in five replicates: 2–4 cm SC, 64–66 cm LC, and 166–168 cm LC. The lyophilized sediments were thoroughly homogenized with mortar and pestle. Then, an aqueous solution (30-µL) containing 10 ng NOD (Alexis Biochemicals, Lausen, Switzerland) in MilliQ water was added to 2.0-g sediment sample. The sample was vortexed for 1 min and left overnight at 4 °C. NOD was extracted with 5 and 2.5 mL of 75% methanol in water (LiChrosolv, gradient grade HPLC, Merck, Darmstadt, Germany) by vortexing (10 min) and bath sonication (1 min). The combined extracts were centrifuged (10,000× *g*; 15 min; 8 °C) and evaporated to dryness. The residue was dissolved in 1 mL of 75% methanol and analyzed with LC-MS/MS system. Every second layer of the two sediment cores (2.0 g) was extracted with 75% methanol following the same procedure.

### 4.3. LC-MC/MS Analysis of Cyanopeptides

Screening for cyanobacterial peptides, including NOD, microcystins (MCs), and anabaenopeptins (APs), was performed with the application of Agilent 1200 HPLC system (Agilent Technologies, Waldboronn, Germany) linked to a hybrid triple quadrupole/linear ion trap mass spectrometer (QTRAP5500, Applied Biosystems, Sciex, Concorde, ON, Canada). Sample components were separated using, a Zorbax Eclipse XDB-C18 column (4.6 × 150 mm; 5 µm) (Agilent Technologies, Santa Clara, CA, USA) and a gradient elution (0.6 mL/min) with a mixture of 5% aqueous acetonitrile (A) and 100% acetonitrile (B), both containing 0.1% formic acid. In the first run, Information Depended Acquisition (IDA) mode was used, as described earlier [13,14,55]. Then, to verify the presence of detected peptides and to determine the concentration of NOD, multiple reaction monitoring experiments were performed. In the method, the following transitions were used: 825 → 135 (quantifier, collision energy CE 80; LOQ = 0.05 ng/g d.w.; S/N > 5), 825 → 227 (CE 65), and 825 → 163 (CE 60) for NOD; 828 → 637, 405, 120, and 84 (at CE 60) for AP827; and 884 → 689, 511, 339, 164, and 107 (at CE 60) for AP883. 

### 4.4. DNA Isolation

For DNA analyses, sediment samples (500 mg) listed in Supplementary Material Table S2 were used. The DNA extractions were performed using two isolation kits: NucleoSpin^®^ Soil (Macherey-Nagel, Düren, Germany) and a FastDNA™ Kit for Soil (MP Biomedicals, Santa Ana, CA, USA). To optimize the procedure, different modifications to manufacturer’s instructions were made (Supplementary Material Table S5). All extractions were performed in two variants: with and without a clean-up using Anty-Inhibitor Kit (A&A Biotechnology, Gdynia, Poland). The experimental variants and their results are presented in Supplementary Material Table S3. The presence of DNA was confirmed using a 1% agarose gel electrophoresis. The quality and quantity of extracted DNA was determined with SpectraMax^®^ i3 Platform (Molecular Devices LLC., Sunnyvale, CA, USA) equipped with SpectraDrop Micro-Volume Microplate. 

### 4.5. Polymerase Chain Reaction (PCR) and Sequence Analysis

Immediately after DNA isolation, the polymerase chain reactions were performed in a Mastercycler^®^ nexus GSX1 (Eppendorf, Hamburg, Germany). For amplification of *mcyE*/*ndaF* gene, the same primers (HEPF and HEPR) and PCR cycling conditions as in Jungblut and Neilan [56] were used. The *ndaF* and *mcyE* genes are orthologous and the primers used in this work amplify both of them. Amplification of the *cpcBA*-IGS with PCβF and PCαR primers was performed according to Neilan et al. [57], but the number of cycles was reduced from 40 to 35. Both PCRs were run in 25 µL solution containing approx. 150 ng of DNA, 5 pmol of each specific oligonucleotide primer, 12.5 µL of MyTaq™ Red Mix (Bioline Reagents Ltd., London, UK) and 1 μg/μL of bovine albumin (Sigma-Aldrich, Saint Louis, MO, USA). DNA isolated from *N. spumigena* strains CCNP1401 and CCY9414 [13] was used as a positive control and MilliQ water as a negative control. The presence of PCR products was checked with 1% agarose gel electrophoresis. Selected PCR products were purified with ExtractMe DNA clean-up kit (Blirt S.A., Gdańsk, Poland) and sequenced (Genomed S.A., Warszawa, Poland). The obtained nucleotide sequences were edited with Chromas Lite 2.1 and compared to the sequences in the NCBI GenBank (http://www.ncbi.nlm.nih.gov) using *blastn* algoritm (http://blast.ncbi.nlm.nih.gov). Nucleotide sequences were deposited in the GenBank database (Supplementary Material Table S1). *N. spumigena* PC-IGS sequences (496 bp) available from GenBank and the sequences of amplicons obtained in this study from sediment samples were aligned in MEGA version 6 [58]. The alignment was corrected manually. Neighbor-joining (NJ), maximum likelihood (ML), and maximum parsimony (MP) phylogenetic trees were constructed. For each tree, bootstrap analysis of 1000 replications was performed. Pairwise distances (p-distances) among sequences were calculated according to Kimura [59] in Mega version 6. 

## 5. Conclusions

We showed that the cyclic non-ribosomal cyanopeptides, as stable and specific markers, can find wide application in environmental studies. Unfortunately, with the limited knowledge on environmental conditions in earlier stages of the Baltic Sea, and without the precise dating of the sediments, a reliable explanation for the unexpectedly high peak in NOD concentrations in 170–180 cm layers is impossible. As the same subpopulations of *N. spumigena* occurred in the Baltic at that time as today, the peak NOD concentrations, significantly exceeding the values recorded in recent sediments, could indicate more abundant blooms of the species. It cannot be excluded that, beside physical and chemical conditions, also some biotic factors have significant effect on toxic *N. spumigena* blooms in the Baltic Sea. However, our knowledge about this kind of interactions is still limited. 

## Supplementary Materials

The following are available online at http://www.mdpi.com/1660-3397/16/4/116/s1. Figure S1: MRM chromatograms of nodularin (NOD) standard (A) and NOD extracted from 284–286 cm layer of long sediment core (LC) collected in the Gulf of Gdańsk, Southern Baltic Sea (B); the MRM transitions are marked in different colors. Chemical structure and enhanced ion product mass spectra (EPI) of nodularin standard (C) and EPI of NOD extracted from 284–286 cm layer of LC (D). Figure S2: MRM chromatogram of anabaenopeptin AP827 extracted from 170–172 cm layer of long sediment core (LC) collected in the Gulf of Gdańsk, Southern Baltic Sea; the MRM transitions are marked in different colors (A). Chemical structure and enhanced ion product mass spectra of anabaenopeptin AP827 extracted from *N. spumigena* CCNP1401 (B) and EPI of AP827 170–172 cm layer of LC (C). Figure S3: MRM chromatogram of anabaenopeptin AP883a extracted from 174–176 cm layer of long sediment core (LC) collected in the Gulf of Gdańsk, Southern Baltic Sea; the MRM transitions are marked in different colors (A). Chemical structure and enhanced ion product mass spectra of anabaenopeptin AP883a extracted from *N. spumigena* CCNP1402 (B), and EPI of AP883a extracted from 174–176 cm layer of LC (C). Figure S4: Neighbour-joining (NJ) (A) and Maximum parsimony (MP) (B) phylogenetic trees based on the *cpcBA*-IGS sequences (496 bp) obtained from DNA isolated from Baltic sediments (marked in blue) and reference *cpcBA*-IGS sequences (retrieved from NCBI) from *N. spumigena* strains (marked in black). Phylogenetic relationships were bootstrapped 1000 times. The branches with less than 50% bootstrap are shown as unresolved. Similar *cpcBA*-IGS sequences are marked as GT_A and GT_B, respectively (GT–genotype). Table S1: Changes in nodularin concentrations [ng/g dw] and changes in relative amounts of anabaenopeptins (expressed as a ratio of AP peak area/g dw) in short (SC) and long core (LC). Table S2: List of sediment samples from short core (SC) and long core (LC) and type of genetic analysis done in the work (+ indicates type of the analysis done with use of selected sediment sample, ++ indicates presence of selected PCR product). Table S3: The quantity (ng/µL) and quality (A_260/280_) of DNA extracted from selected sediment layers. (MP–DNA isolated with FastDNA™ Kit for Soil, MPA–DNA isolated with FastDNA™ Kit for Soil and cleaned-up with Anty-Inhibitor Kit, N1–DNA isolated with NucleoSpin^®^ Soil using SL1 buffer, N1A–DNA isolated with NucleoSpin^®^ Soil using SL1 buffer and cleaned-up with Anty-Inhibitor Kit, N2–DNA isolated with NucleoSpin^®^ Soil using SL2 buffer, N2A–DNA isolated with NucleoSpin^®^ Soil using SL2 buffer and cleaned-up with Anty-Inhibitor Kit). Table S4: Exemplary studies on the presence and diversity of cyanobacterial communities in sediment samples conducted with the application of genetic methods. * The oldest analyzed cyanobacterial DNA. Table S5: Modifications made to manufactures instructions during the isolation of DNA with two different kits.

## References

[1] Bianchi T.S., Westman P., Andrén T., Rolff C., Elmgren R. (2000). Cyanobacterial blooms in the Baltic Sea: Natural or human-induced?. Limnol. Oceanogr..

[2] Voss M., Kowalewska G., Brenner W. (2001). Microfossil and biogeochemical indicators of environmental changes in the Gotland Deep during the last 10.000 years. Baltica.

[3] Funkey C.P., Conley D.J., Reuss N.S., Humborg C., Jilbert T., Slomp C.P. (2014). Hypoxia sustains cyanobacteria blooms in the Baltic Sea. Environ. Sci. Technol..

[4] Briggs D.E.G., Summons R.E. (2014). Ancient biomolecules: Their origins, fossilization, and role in revealing the history of life. Bioessa.

[5] Finni T., Kononen K., Olsonen R., Wallström K. (2001). The history of cyanobacterial blooms in the Baltic Sea. Ambio.

[6] Hübel H., Hübel M. (1974). Nitrogen-fixation in the coastal waters of the Baltic Sea. Z. Allg. Mikrobiol..

[7] Sivonen K., Kononen K., Carmichael W.W., Dahlem A.M., Rinehart K.L., Kiviranta J., Niemelä S.I. (1989). Occurrence of the hepatotoxic cyanobacterium *Nodularia spumigena* in the Baltic Sea and structure of the toxin. Appl. Environ. Microbiol..

[8] Ohta T., Sueoka E., Iida N., Komori A., Suganuma M., Nishiwaki R., Tatematsu M., Kim S.J., Carmichael W.W., Fujiki H. (1994). Nodularin, a potent inhibitor of protein phosphatases 1 and 2A, is a new environmental carcinogen in male F344 rat liver. Cancer Res..

[9] Fujiki H., Suganuma M. (2011). Tumor promoters–microcystin-LR, nodularin and TNF-α and human cancer development. Anticancer Agents Med. Chem..

[10] Kankaanpää H.T., Sjövall O., Huttunen M., Olin M., Karlsson K., Hyvärinen K., Sneitz L., Härkönen J., Sipiä V.O., Meriluoto J.A. (2009). Production and sedimentation of peptide toxins nodularin-R and microcystin-LR in the northern Baltic Sea. Environ. Pollut..

[11] Fewer D.P., Jokela J., Paukku E., Österholm J., Wahlsten M., Permi P., Rouhiainen L., Gomez-Saez G.V., Sivonen K. (2013). New structural variants of aeruginosin produced by the toxic bloom forming cyanobacterium *Nodularia spumigena*. PLoS ONE.

[12] Liu L., Budnjo A., Jokela J., Haug B.E., Fewer D.P., Wahlsten M., Rouhiainen L., Permi P., Fossen T., Sivonen K. (2015). Pseudoaeruginosins, nonribosomal peptides in *Nodularia spumigena*. ACS Chem. Biol..

[13] Mazur-Marzec H., Bertos-Fortis M., Toruńska-Sitarz A., Fidor A., Legrand C. (2016). Chemical and Genetic Diversity of *Nodularia spumigena* from the Baltic Sea. Mar. Drugs.

[14] Spoof L., Błaszczyk A., Meriluoto J., Cegłowska M., Mazur-Marzec H. (2016). Structures and activity of new anabaenopeptins produced by Baltic Sea cyanobacteria. Mar. Drugs.

[15] Kaasalainen U., Fewer D.P., Jokela J., Wahlsten M., Sivonen K., Rikkinen J. (2012). Cyanobacteria produce a high variety of hepatotoxic peptides in lichen symbiosis. Proc. Natl. Acad. Sci. USA.

[16] Gehringer M.M., Adler L., Roberts A.A., Moffitt M.C., Mihali T.K., Mills T.J.T., Fieker C., Neilan B.A. (2012). Nodularin, a cyanobacterial toxin, is synthesized in planta by symbiotic *Nostoc* sp.. ISME J..

[17] Jokela J., Heinilä L.M.P., Shishido T.K., Wahlsten M., Fewer D.P., Fiore M.F., Wang H., Haapaniemi E., Permi P., Sivonen K. (2017). Production of high amounts of hepatotoxin nodularin and new protease inhibitors pseudospumigins by the Brazilian benthic *Nostoc* sp. CENA543. Front. Microbiol..

[18] McGregor G.B., Sendall B.C. (2017). *Iningainema pulvinus* gen nov., sp nov. (Cyanobacteria, Scytonemataceae) a new nodularin producer from Edgbaston Reserve, north-eastern Australia. Harmful Algae.

[19] Szymczak-Żyła M., Kowalewska G. (2009). Chloropigments in sediments of the Gulf of Gdańsk deposited during the last 4000 years as indicators of eutrophication and climate change. Palaeoecology.

[20] Okano K., Maseda H., Sugita K., Saito T., Utsumi M., Maekawa T., Kobayashi M., Sugiura N. (2006). Biochemical characteristics of microcystin-LR degradation by typical protease. Jpn. J. Water Treat. Biol..

[21] Kormas K.A., Lymperopoulou D.S. (2013). Cyanobacterial toxin degrading bacteria: Who are they?. Biomed. Res. Int..

[22] Harada K.I., Tsuji K., Watanabe M.F., Kondo F. (1996). Stability of microcystins from cyanobacteria—III. Effect of pH and temperature. Phycologia.

[23] Metcalf J.S., Codd G.A. (2000). Microwave oven and boiling waterbath extraction of hepatotoxins from cyanobacterial cells. FEMS Microbiol. Lett..

[24] Mazur H., Pliński M. (2001). Stability of cyanotoxins, microcystin-LR, microcystin-RR and nodularin in seawater and BG-11 medium of different salinity. Oceanologia.

[25] Zhang D., Xie P., Chen J. (2010). Effect of temperature on the stability of microcystins in muscle of fish and its consequences for food safety. Bull. Environ. Contam. Toxicol..

[26] Wiedner C., Visser P.M., Fastner J., Metcalf J.S., Codd G.A., Mur L.R. (2003). Effects of light on the microcystin content of Microcystis strain PCC 7806. Appl. Environ. Microbiol..

[27] Panieri G., Lugli S., Manzi V., Roveri M., Schreiber B.C., Palińska K.A. (2010). Ribosomal RNA gene fragments from fossilized cyanobacteria identified in primary gypsum from the late Miocene, Italy. Geobiology.

[28] Lyra K., Sinkko H., Rantanen M., Paulin L., Kotilainen A. (2013). Sediment bacterial communities reflect the history of a Sea Basin. PLoS ONE.

[29] Webster G., Newberry C.J., Fry J.C., Weightman A.J. (2003). Assessment of bacterial community structure in the deep sub-seafloor biosphere by 16S rDNA-based techniques: A cautionary tale. J. Microbiol. Methods.

[30] Bowman J.P., Rea S.M., McCammon S.A., McMeekin T.A. (2000). Diversity and community structure within anoxic sediment from marine salinity meromictic lakes and a coastal meromictic marine basin, Vestfold Hills, Eastern Antarctica. Environ. Microbiol..

[31] Dadheech P.K., Krienitz L., Kotut K., Ballot A., Casper P. (2009). Molecular detection of uncultured cyanobacteria and aminotransferase domains for cyanotoxin production in sediments of different Kenyan lakes. FEMS Microbiol. Ecol..

[32] Rinta-Kanto J.M., Saxton M.A., DeBruyn J.M., Smith J.L., Marvin C.H., Krieger K.A., Sayler G.S., Boyer L.G., Wilhelm S.W. (2009). The diversity and distribution of toxigenic *Microcystis* spp. in present day and archived pelagic and sediment samples from Lake Erie. Harmful Algae.

[33] Savichtcheva O., Debroas D., Kurmayer R., Villar C., Jenny J.P., Arnaud F., Perga M.E., Domaizon I. (2011). Quantitative PCR enumeration of total/toxic *Planktothrix rubescens* and total cyanobacteria in preserved DNA isolated from lake sediments. Appl. Environ. Microbiol..

[34] Domaizon I., Savichtcheva O., Debroas D., Arnaud F., Villar C., Pignol C., Alric B., Perga M.E. (2013). DNA from lake sediments reveals the long-term dynamics and diversity of *Synechococcus* assemblages. Biogeosciences.

[35] De La Escalera M.G., Antoniades D., Bonilla S., Piccini C. (2014). Application of ancient DNA to the reconstruction of past microbial assemblages and for the detection of toxic cyanobacteria in subtropical freshwater ecosystems. Mol. Ecol..

[36] Monchamp M.E., Walser J.C., Pomati F., Spaak P. (2016). Sedimentary DNA reveals cyanobacterial community diversity over 200 years in two perialpine lakes. Appl. Environ. Microbiol..

[37] Kyle M., Haande S., Sřnstebř J., Rohrlack T. (2015). Amplification of DNA in sediment cores to detect historic *Planktothrix* occurrence in three Norwegian lakes. J. Paleolimnol..

[38] Pal S., Gregory-Eaves I., Pick F.R. (2015). Temporal trends in cyanobacteria revealed through DNA and pigment analyses of temperate lake sediment cores. J. Paleolimnol..

[39] Fernandez-Carazomailto R., Verleyen E., Hodgson D.A., Roberts S.J., Waleron K., Vyverman W., Wilmotte A. (2013). Late Holocene changes in cyanobacterial community structure in maritime Antarctic lakes. J. Paleolimnol..

[40] Hou W., Dong H., Li G., Yang J., Coolen M.J., Liu X., Wang S., Jiang H., Wu X., Xiao H. (2014). Identification of photosynthetic plankton communities using sedimentary ancient DNA and their response to late-Holocene climate change on the Tibetan Plateau. Sci. Rep..

[41] Coolen M.J.L., Talbot H.M., Abbas B.A., Ward C., Schouten S., Volkman J.K., Damsté J.S.S. (2008). Sources for sedimentary bacteriohopanepolyols as revealed by 16S rDNA stratigraphy. Environ. Microbiol..

[42] Li G., Dong H., Hou W., Wang S., Jiang H., Yang J., Wu G. (2016). Temporal succession of ancient phytoplankton community in Qinghai Lake and implication for paleo-environmental change. Sci. Rep..

[43] Barker G.L.A., Hayes P.K., O’Mahony S.L., Vacharapiyasophon P., Walsby A.E. (1999). A molecular and phenotypic analysis of *Nodularia* (cyanobacteria) from the Baltic Sea. J. Phycol..

[44] Laamanen M.J., Gugger M.F., Lehtimäki J., Haukka K., Sivonen K. (2001). Diversity of toxic and non-toxic *Nodularia* isolates (Cyanobacteria) and filaments from the Baltic Sea. Appl. Environ. Microbiol..

[45] Janson S., Granéli E. (2002). Phylogenetic analyses of nitrogen-fixing cyanobacteria from Baltic Sea reveal sequence anomalies in the phycocyanin operon. Int. J. Syst. Evol. Microbiol..

[46] Rounge T.B., Rohrlack T., Decenciere B., Edvardsen B., Kristensen T., Jakobsen K.S. (2010). Subpopulation differentiation associated with nonribosomal peptide synthetase gene cluster dynamics in the cyanobacterium *Planktothrix* spp.. J. Phycol..

[47] Sønstebø J.H., Rohrlack T. (2011). Possible implication of chytrid parasitism for population subdivision in freshwater cyanobacteria of the genus *Planktothrix*. Appl. Environ. Microbiol..

[48] Sogge H., Rohrlack T., Rounge T.B., Sønstebe J.H., Tooming-Klunderud A., Kristensen T., Jakobsen K.S. (2013). Gene flow, recombination, and selection in cyanobacteria: Population structure of geographically related *Planktothrix* freshwater strains. Appl. Environ. Microbiol..

[49] Lehtimäki J., Moisander P., Sivonen K., Kononen K. (1997). Growth, nitrogen fixation, and nodularin production by two Baltic Sea cyanobacteria. Appl. Environ. Microbiol..

[50] Hobson P., Fallowfield H. (2003). Effect of irradiance, temperature and salinity on growth and toxin production by *Nodularia spumigena*. Hydrobiologia.

[51] Wang T., Surge D., Mithen S. (2012). Seasonal temperature variability of the Neoglacial (3300-2500 BP) and Roman Warm Period (2500-1600 BP) reconstructed from oxygen isotope ratios of limpet shells (*Patella vulgata*), Northwest Scotland. Palaeogeogr. Palaeoclimatol. Palaeoecol..

[52] Büntgen U., Myglan V.S., Ljungqvist F.C., McCormick M., Di Cosmo N., Sigl M., Jungclaus J., Wagner S., Krusic P.J., Esper J. (2016). Cooling and societal change during the Late Antique Little Ice Age from 536 to around 660 AD. Nat. Geosci..

[53] Kabel K., Moros M., Porsche C., Neumann T., Adolphi F., Andersen T.J., Siegel H., Gerth M., Leipe T., Jansen A., Damsté J.S.S. (2012). Impact of climate change on the Baltic Sea ecosystem over the past 1000 years. Nat. Clim. Chang..

[54] O’Neil J.M., Davis T.W., Burford M.A., Gobler C.J. (2012). The Rise of Harmful Cyanobacteria Blooms: Potential Role of Eutrophication and Climate Change. Harmful Algae.

[55] Šulčius S., Pilkaityte R., Mazur-Marzec H., Kasperovičiene J., Ezhova E., Błaszczyk A., Paškauskas R. (2015). Increased risk of exposure to microcystins in the scum of the filamentous cyanobacterium *Aphanizomenon flos-aquae* accumulated on the western shoreline of the Curonian Lagoon. Mar. Poll. Bull..

[56] Jungblut A.D., Neilan B.A. (2006). Molecular identification and evolution of the cyclic peptide hepatotoxins, microcystin and nodularin, synthetase genes in three orders of cyanobacteria. Arch. Microbiol..

[57] Neilan B.A., Jacobs D., Goodman A.E. (1995). Genetic diversity and phylogeny of toxic cyanobacteria determined by DNA polymorphisms within the phycocyanin locus. Appl. Environ. Microbiol..

[58] Tamura K., Stecher G., Peterson D., Filipski A., Kumar S. (2013). MEGA6: Molecular evolutionary genetics analysis version 6.0. Mol. Biol. Evol..

[59] Kimura M. (1980). A simple method for estimating evolutionary rates of base substitutions through comparative studies of nucleotide sequences. J. Mol. Evol..

