# Supplementary material for: Specific Chemical and Genetic Markers Revealed a Thousands-Year Presence of Toxic Nodularia spumigena in the Baltic Sea
Source: Mar Drugs. 2018 Apr 4;16(4):116. doi: 10.3390/md16040116 (PMC5923403; doi:10.3390/md16040116)
Supplement: Supplementary file 1 [file marinedrugs-16-00116-s001.pdf]

# **Supplementary Material**: Specific Chemical and Genetic Markers Revealed a Thousands-Year Presence of Toxic *Nodularia spumigena* in the Baltic Sea

**Marta Cegłowska[1], Anna Toruńska-Sitarz[2], Grażyna Kowalewska[1], Hanna Mazur-Marzec[1,2]***

[1] Institute of Oceanology, Polish Academy of Sciences, Powstańców Warszawy 55, PL-81-727 Sopot, Poland, mceglowska@iopan.pl (M.C.), kowalewska@iopan.gda.pl (G.K.)
[2] University of Gdańsk, Faculty of Oceanography and Geography, Division of Marine Biotechnology, Marszałka J. Płisudskiego 46, PL-81-378 Gdynia, Poland, anna.torunska@ug.edu.pl (A.T-S.)
* Correspondence:biohm@ug.edu.pl; Tel.: +48-58-523-66-21

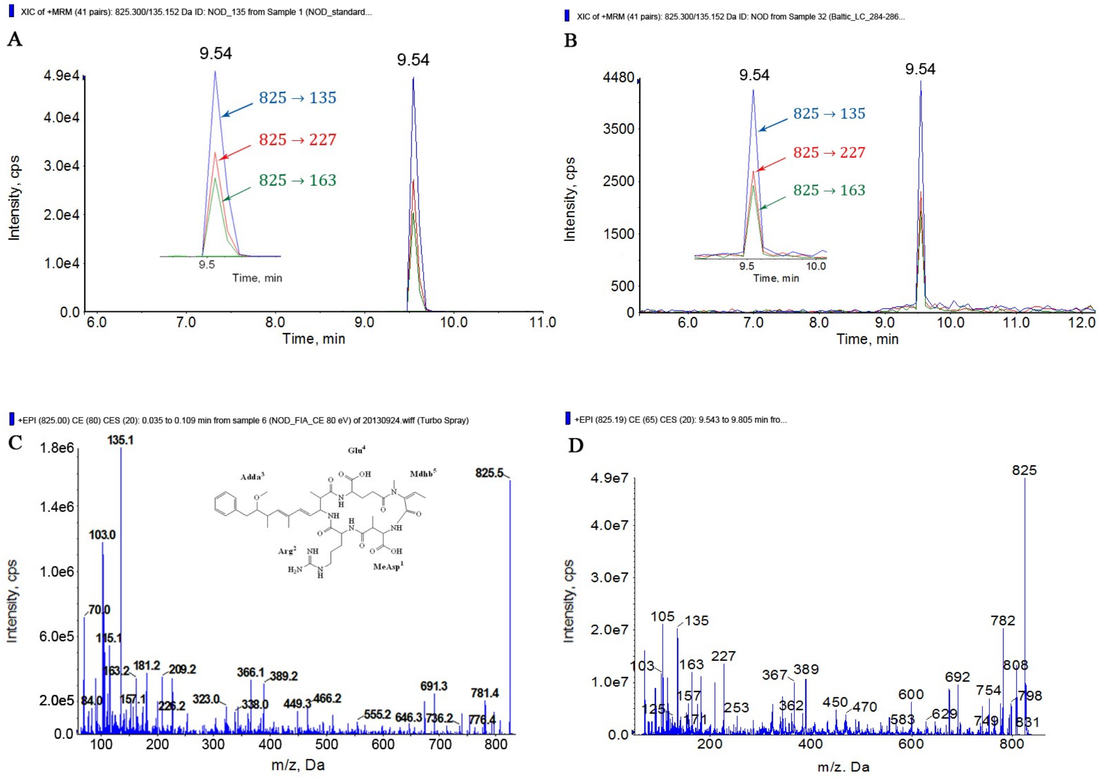


**Figure S1:** MRM chromatograms of nodularin (NOD) standard (A) and NOD extracted from 284–286 cm layer of long sediment core (LC) collected in the Gulf of Gdańsk, Southern Baltic Sea (B); the MRM transitions are marked in different colors. Chemical structure and enhanced ion product mass spectra (EPI) of nodularin standard (C) and EPI of NOD extracted from 284–286 cm layer of LC (D).

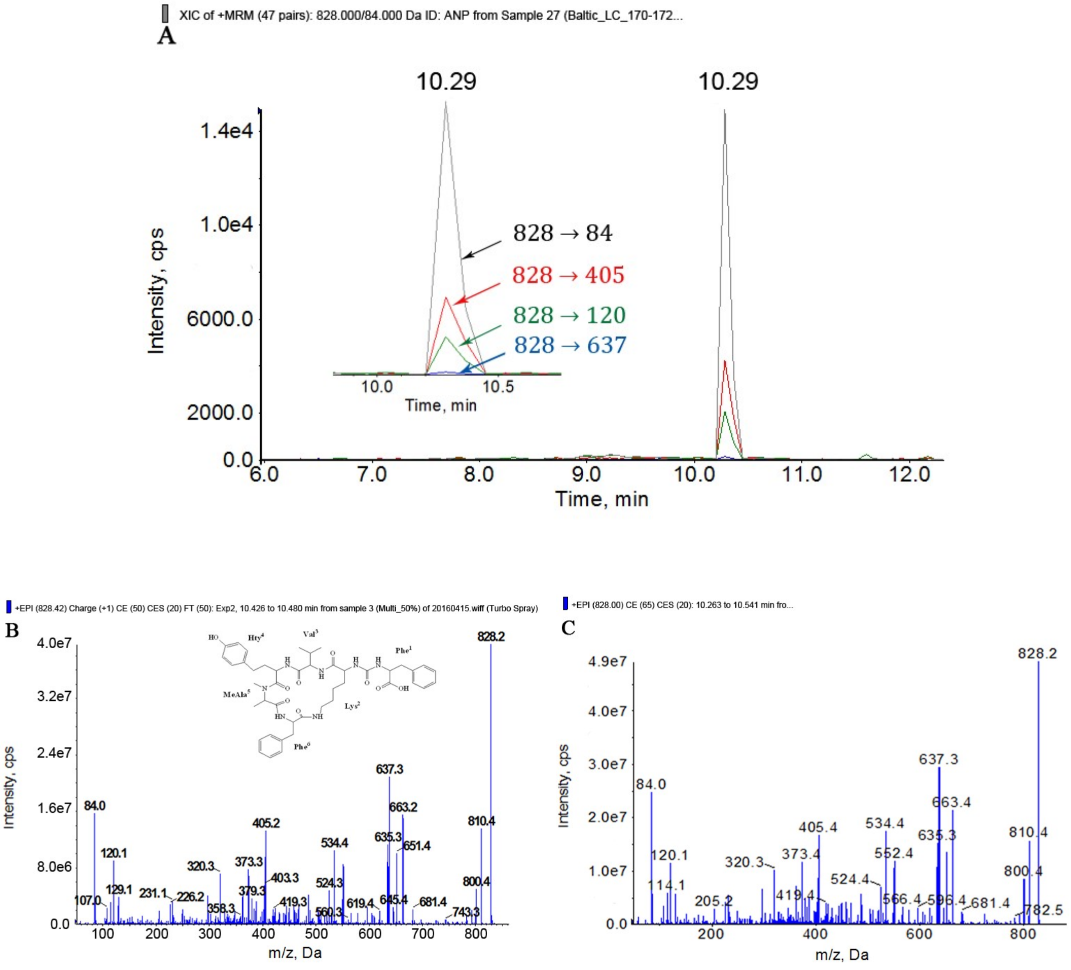


**Figure S2:** MRM chromatogram of anabaenopeptin AP827 extracted from 170–170 cm layer of long sediment core (LC) collected in the Gulf of Gdańsk, Southern Baltic Sea; the MRM transitions are marked in different colors (A). Chemical structure and enhanced ion product mass spectra of anabaenopeptin AP827 extracted from *N. spumigena* CCNP1401 (B) and EPI of AP827 170–170 cm layer of LC (C).

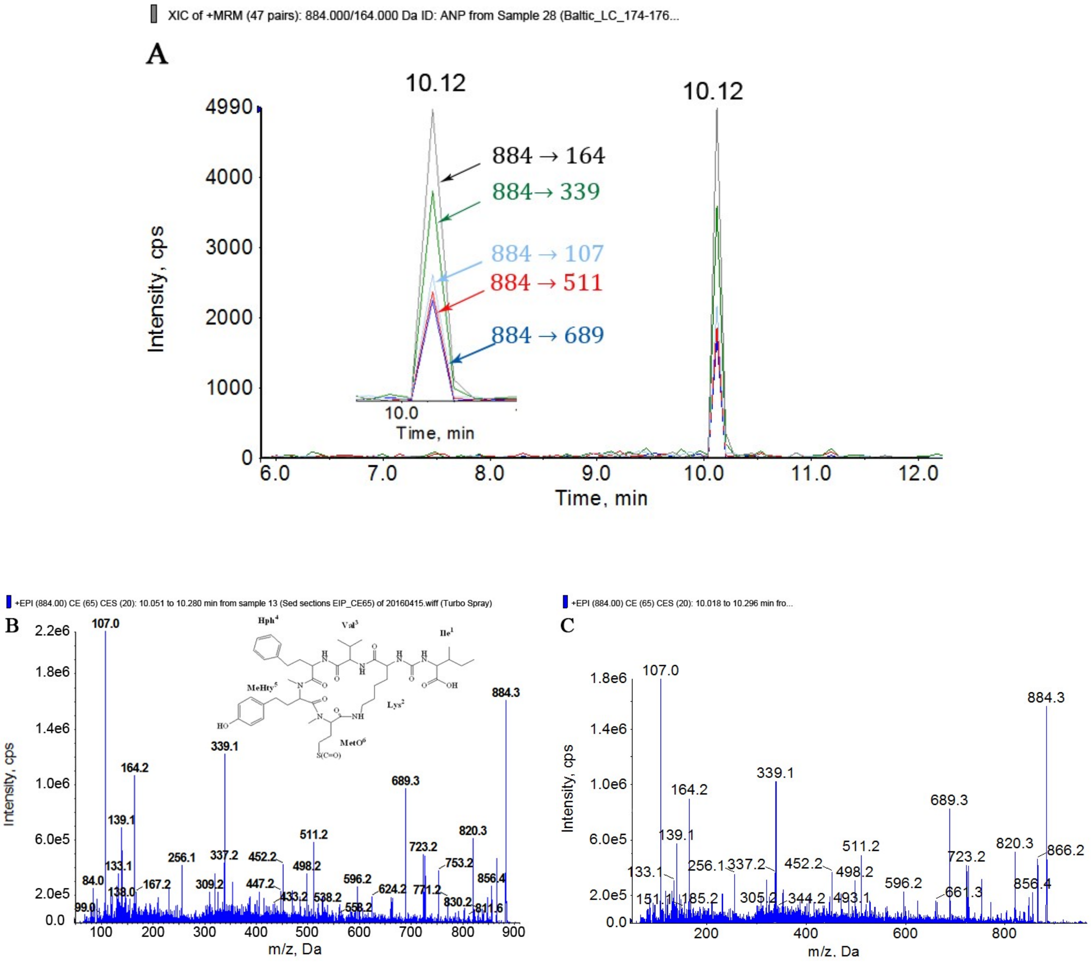


**Figure S3:** MRM chromatogram of anabaenopeptin AP883a extracted from 174–176 cm layer of long sediment core (LC) collected in the Gulf of Gdańsk, Southern Baltic Sea; the MRM transitions are marked in different colors (A). Chemical structure and enhanced ion product mass spectra of anabaenopeptin AP883a extracted from *N. spumigena* CCNP1402 (B), and EPI of AP883a extracted from 174–176 cm layer of LC (C).

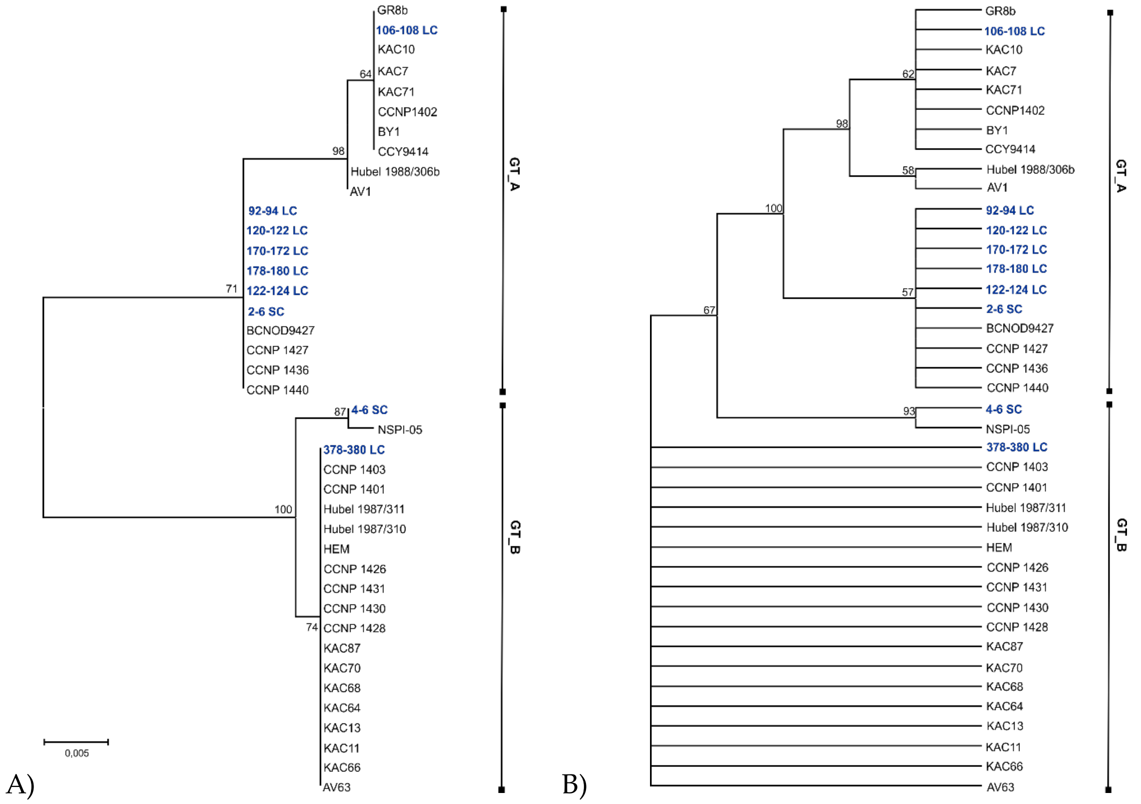


**Figure S4:** Neighbour-joining (NJ) (A) and Maximum parsimony (MP) (B) phylogenetic trees based on the *cpcBA*-IGS sequences (496 bp) obtained from DNA isolated from Baltic sediments (marked in blue) and reference *cpcBA*-IGS sequences (retrieved from NCBI) from *N. spumigena* strains (marked in black). Phylogenetic relationships were bootstrapped 1000 times. The branches with less than 50% bootstrap are shown as unresolved. Similar *cpcBA*-IGS sequences are marked as GT A and GT B, respectively (**GT**, genotype).

**Table S1:** Changes in nodularin concentrations [ng/g dw] and changes in relative amounts of anabaenopeptins (expressed as a ratio of AP peak area/g dw) in short (SC) and long core (LC).

| Core layer | NOD concentration [ng/g] | AP827 | AP883a |
|---|---|---|---|
| | | [peak area/g] | |
| **Short core (SC)** | | | |
| 0–2 | 1.05 | 0 | 0.36 |
| 2–4 | 1.63 | 0.72 | 0.44 |
| 4–6 | 4.32 | 0.76 | 0.36 |
| 6–8 | 1.45 | 0.25 | 0.54 |
| 12–14 | 2.47 | 0 | 0.83 |
| 14–16 | 0.12 | 0.2 | 0 |
| 16–18 | 0.18 | 0 | 0 |
| 18–20 | 0.13 | 0.5 | 0 |
| 20–22 | 0.11 | 0.4 | 0 |
| 22–24 | 0.14 | 0.16 | 0 |
| 24–26 | 0.27 | 0.27 | 0 |
| 26–28 | 0.38 | 0.27 | 0 |
| 28–30 | 0.12 | 0.28 | 0 |
| **Long core (LC)** | | | |
| 0–2 | 0.23 | 0.13 | 0 |
| 2–4 | 0.15 | 0.11 | 0 |
| 4–6 | 0.23 | 0.28 | 0 |
| 6–8 | 0.21 | 0.24 | 0 |
| 8–10 | 0.24 | 0.39 | 0.12 |
| 10–12 | 0.26 | 0.27 | 0 |
| 12–14 | 0.23 | 0.27 | 0 |
| 14–16 | 0.30 | 0.39 | 0.23 |
| 16–18 | 0.33 | 0.36 | 0.21 |
| 18–20 | 0.33 | 0.26 | 0.29 |
| 20–22 | 0.34 | 0.47 | 0.29 |
| 24–26 | 0.33 | 0.36 | 0.13 |
| 28–30 | 0.32 | 0.41 | 0.11 |
| 32–34 | 0.63 | 0.82 | 0.6 |
| 36–38 | 0.67 | 0.72 | 0.42 |
| 40–42 | 0.73 | 0.93 | 0.56 |
| 44–46 | 0.68 | 1.04 | 0.52 |
| 48–50 | 0.68 | 0.67 | 0.3 |
| 52–54 | 0.74 | 0.71 | 0.44 |
| 56–58 | 0.62 | 0.76 | 0.29 |
| 60–62 | 0.61 | 0.74 | 0.42 |
| 64–66 | 0.61 | 0.73 | 0.29 |
| 68–70 | 0.55 | 0.58 | 0.46 |
| 72–74 | 0.61 | 0.64 | 0.39 |
| 76–78 | 0.58 | 0.72 | 0.4 |
| 78–80 | 0.74 | 1.25 | 0.19 |
| 80–82 | 2.52 | 1.66 | 1.61 |
| 82–84 | 4.84 | 16.2 | 1.42 |
| 84–86 | 1.68 | 2.82 | 1.27 |
| 86–88 | 8.96 | 6.01 | 1 |
| 88–90 | 2.83 | 3.85 | 1.94 |
| 90–92 | 29.98 | 31.4 | 6.68 |
| 92–94 | 1.87 | 2.33 | 1.38 |
| 94–96 | 1.86 | 0.93 | 0 |
| 96–98 | 2.08 | 1.72 | 1.5 |
| 98–100 | 13.48 | 13.95 | 1.05 |
| 100–102 | 1.92 | 1.73 | 1.43 |
| 102–104 | 1.29 | 1.56 | 0.38 |
| 104–106 | 1.56 | 1.24 | 0.91 |
| 106–108 | 31.44 | 24.62 | 6.66 |
| 108–110 | 1.27 | 0.98 | 0.79 |
| 110–112 | 1.09 | 0.98 | 1.01 |
| 112–114 | 1.35 | 2.14 | 0.93 |
| 114–116 | 10.13 | 6.91 | 3.91 |
| 116–118 | 1.42 | 1.45 | 1 |
| 118–120 | 7.10 | 10.22 | 2.03 |
| 120–122 | 7.83 | 1.43 | 1.07 |
| 122–124 | 31.31 | 23.49 | 4.86 |
| 124–126 | 1.24 | 1.12 | 0.86 |
| 126–128 | 0.55 | 1.26 | 0 |
| 128–130 | 0.64 | 0.81 | 1.93 |
| 130–132 | 1.94 | 12.29 | 4.08 |
| 132–134 | 1.71 | 1.34 | 2.52 |
| 134–136 | 1.13 | 0.53 | 4.3 |
| 136–138 | 1.29 | 1.53 | 2.97 |
| 138–140 | 3.00 | 3.06 | 1.26 |
| 140–142 | 0.99 | 1.06 | 1.07 |
| 142–144 | 1.04 | 1.11 | 0 |
| 144–146 | 0.70 | 0.95 | 0.35 |
| 146–148 | 0.35 | 0.11 | 0 |
| 148–150 | 1.24 | 1.67 | 1.06 |
| 150–152 | 0.48 | 0.14 | 0 |
| 152–154 | 0.27 | 0.74 | 0.2 |
| 154–156 | 0.51 | 0.5 | 0 |
| 156–158 | 0.34 | 0.5 | 0 |
| 158–160 | 0.96 | 1.97 | 0.87 |

| Core layer | NOD concentration [ng/g] | AP827 | AP883a |
| --- | --- | --- | --- |
| | | [peak area/g] | |
| Long core (LC) | | | |
| 160–162 | 0.46 | 1.02 | 0.12 |
| 162–164 | 0.21 | 0 | 0 |
| 164–166 | 0.46 | 0.61 | 0.33 |
| 166–168 | 0.91 | 1.09 | 0 |
| 168–170 | 0.31 | 0.97 | 0.15 |
| 170–172 | 225.04 | 67.77 | 13.83 |
| 172–174 | 0.52 | 0.6 | 0.53 |
| 174–176 | 185.00 | 57.07 | 20.6 |
| 176–178 | 0.41 | 1.17 | 1.06 |
| 178–180 | 49.71 | 56.4 | 4.82 |
| 180–182 | 5.95 | 0.83 | 1.12 |
| 182–184 | 3.42 | 2.78 | 0 |
| 184–186 | 0.30 | 0.98 | 0.73 |
| 186–188 | 0.39 | 0 | 0 |
| 188–190 | 0.16 | 0 | 0 |
| 192–194 | 0.15 | 2.8 | 0 |
| 196–198 | 0.19 | 0.19 | 0 |
| 202–204 | 0.24 | 0.16 | 0 |
| 206–208 | 0.14 | 0.14 | 0 |
| 210–212 | 0.27 | 0.27 | 0.1 |
| 214–216 | 0.25 | 0.14 | 0.14 |
| 218–220 | 0.33 | 0.26 | 0 |
| 222–224 | 0.24 | 0 | 0 |
| 226–228 | 0.16 | 0.22 | 0 |
| 230–232 | 0.15 | 0.15 | 0 |
| 234–236 | 0.13 | 0.22 | 0 |
| 238–240 | 0.11 | 0.17 | 0.11 |
| 242–244 | 0.16 | 0.18 | 0 |
| 246–248 | 0.18 | 0.19 | 0 |
| 250–252 | 0.24 | 0.37 | 0 |
| 254–256 | 0.27 | 0.33 | 0.14 |
| 256–258 | 0.28 | 0.23 | 0.14 |
| 260–262 | 0.31 | 0.28 | 0 |
| 264–266 | 0.46 | 0.48 | 0.23 |
| 268–270 | 0.41 | 0.52 | 0.12 |
| 272–274 | 0.31 | 0.28 | 0.24 |
| 276–278 | 0.35 | 0.45 | 0.2 |
| 280–282 | 0.26 | 0.28 | 0.21 |
| 284–286 | 0.26 | 0.73 | 0.43 |
| 288–290 | 0.09 | 0.13 | 0 |
| 292–294 | 0.15 | 0.17 | 0 |
| 296–298 | 0.16 | 0.28 | 0.19 |
| 300–302 | 0.13 | 0.57 | 0 |
| 304–306 | 0.12 | 0.12 | 0 |
| 308–310 | 0.10 | 0.21 | 0 |
| 312–314 | 0.13 | 0.27 | 0 |
| 316–318 | 0.12 | 0.22 | 0 |
| 320–322 | 0.12 | 0.14 | 0 |
| 324–326 | 0.11 | 0.35 | 0 |
| 328–330 | 0.13 | 0.18 | 0 |
| 332–334 | 0.14 | 0.2 | 0 |
| 336–338 | 0.16 | 0.16 | 0 |
| 340–342 | 0.12 | 0.23 | 0 |
| 344–346 | 0.08 | 0.21 | 0.11 |
| 348–350 | 0.07 | 0.2 | 0 |
| 352–354 | 0.10 | 0.25 | 0 |
| 354–356 | 0.11 | 0 | 0 |
| 356–358 | 0.13 | 0.32 | 0 |
| 358–360 | 0.13 | 0.54 | 0.21 |
| 360–362 | 0.10 | 0.32 | 0 |
| 364–366 | 0.11 | 0.26 | 0 |
| 368–370 | 0.12 | 0.33 | 0 |
| 372–374 | 0.16 | 0.32 | 0 |
| 376–378 | 0.12 | 0.33 | 0 |
| 380–382 | 0.14 | 0.4 | 0 |

**Table S2:** List of sediment samples from short core (SC) and long core (LC) and type of genetic analysis done in the work (+ indicates type of the analysis done with use of selected sediment sample, ++ indicates presence of selected PCR product).

| Sediment layer [cm] | PCR (*nda*F/*mcy*E) | | PCR (PC-IGS) | | PC-IGS sequences accession numbers |
|---|---|---|---|---|---|
| | analyzed | detected | analyzed | detected | |
| **Short core (SC)** | | | | | |
| 0–2 | + | ++ | + | ++ | |
| 2–6 | | | + | ++ | MF101236 |
| 4–6 | + | ++ | + | ++ | MF101243 |
| **Long core (LC)** | | | | | |
| 2–4 | + | ++ | + | ++ | |
| 4–6 | + | ++ | + | ++ | |
| 6–8 | + | ++ | + | ++ | |
| 10–12 | + | ++ | + | ++ | |
| 12–14 | + | ++ | + | ++ | |
| 14–16 | + | ++ | + | ++ | |
| 84–86 | | | + | ++ | |
| 92–94 | | | + | ++ | MF101240 |
| 106–108 | | | + | ++ | MF101241 |
| 114–116 | | | + | ++ | |
| 120–122 | | | + | ++ | MF101242 |
| 122–124 | | | + | ++ | MF101237 |
| 124–126 | | | + | ++ | |
| 136–138 | + | | + | ++ | |
| 138–140 | + | | + | ++ | |
| 166–168 | + | | + | ++ | |
| 170–172 | | | + | ++ | MF101239 |
| 178–180 | + | ++ | + | ++ | MF101238 |
| 340–342 | + | | + | ++ | |
| 378–380 | + | | + | ++ | MF101243 |

**Table S3:** The quantity (ng/µL) and quality ($A_{260/280}$) of DNA extracted from selected sediment layers. (**MP** - DNA isolated with FastDNA™ Kit for Soil, **MPA** - DNA isolated with FastDNA™ Kit for Soil and cleaned-up with Anty-Inhibitor Kit, **N1** - DNA isolated with NucleoSpin® Soil using SL1 buffer, **N1A** - DNA isolated with NucleoSpin® Soil using SL1 buffer and cleaned-up with Anty-Inhibitor Kit, **N2** - DNA isolated with NucleoSpin® Soil using SL2 buffer, **N2A** - DNA isolated with NucleoSpin® Soil using SL2 buffer and cleaned-up with Anty-Inhibitor Kit).

| | | | | | | | | | | | | | | |
|---|---|---|---|---|---|---|---|---|---|---|---|---|---|---|
| | **DNA amounts [ng/µL]** | | | | | | | | | | | | | |
| | **Sediment layer [cm]** | | | | | | | | | | | | | |
| **Method** | **0–2** | **4–6** | **2–4** | **4–6** | **6–8** | **10–12** | **12–14** | **14–16** | **136–138** | **138–140** | **166–168** | **178–180** | **340–342** | **378–380** |
| | **Short core (SC)** | | **Long core (LC)** | | | | | | | | | | | |
| MP | 266 | 202 | 92 | 80 | 88 | 110 | 68 | 70 | 64 | 93 | 56 | 37 | 24 | 35 |
| MPA | 67 | 50 | 24 | 18 | 27 | 37 | 43 | 33 | 23 | 36 | 27 | 15 | 5 | 7 |
| N1 | 184 | 119 | 62 | 84 | 48 | 49 | 16 | 43 | 59 | 34 | 30 | 13 | | 16 |
| N1A | 100 | 84 | 30 | 38 | 36 | 315 | 29 | 32 | 17 | 15 | 5 | | | |
| N2 | 225 | 137 | 6 | 23 | 12 | 19 | 17 | 22 | 38 | 25 | 22 | 8 | | |
| N2A | 125 | 104 | | 4 | 4 | 11 | 3 | 7 | 11 | 10 | 7 | | | |

| | | | | | | | | | | | | | | |
|---|---|---|---|---|---|---|---|---|---|---|---|---|---|---|
| | **$A_{260/280}$** | | | | | | | | | | | | | |
| | **Sediment layer [cm]** | | | | | | | | | | | | | |
| **Method** | **0–2** | **4–6** | **2–4** | **4–6** | **6–8** | **10–12** | **12–14** | **14–16** | **136–138** | **138–140** | **166–168** | **178–180** | **340–342** | **378–380** |
| | **Short core (SC)** | | **Long core (LC)** | | | | | | | | | | | |
| MP | 1.6 | 1.5 | 1.4 | 1.5 | 1.5 | 1.5 | 1.6 | 1.6 | 1.6 | 1.6 | 1.6 | 1.8 | 2.2 | 2.0 |
| MPA | 1.4 | 1.3 | 1.5 | 1.7 | 1.8 | 1.5 | 1.8 | 1.7 | 1.4 | 1.6 | 1.6 | 1.8 | 2.0 | 1.8 |
| N1 | 1.9 | 1.9 | 1.8 | 1.6 | 1.9 | 1.8 | 1.7 | 1.7 | 1.4 | 1.6 | 1.5 | 1.3 | | 1.8 |
| N1A | 1.8 | 1.6 | 1.7 | 1.7 | 1.7 | 1.6 | 1.9 | 1.8 | 1.4 | 1.6 | 1.6 | 1.3 | | |
| N2 | 1.7 | 1.6 | 1.5 | 1.2 | 1.2 | 1.1 | 1.4 | 1.1 | 1.6 | 1.6 | 1.2 | 1.3 | | |
| N2A | 1.8 | 1.8 | | 1.2 | 1.2 | 1.2 | 1.5 | 1.1 | 1.6 | 1.6 | 1.2 | | | |

**Table S4:** Exemplary studies on the presence and diversity of cyanobacterial communities in sediment samples conducted with the application of genetic methods. * The oldest analyzed cyanobacterial DNA.

| Type of material | Core length [m] | Estimated age of the deepest part of the core [years] | Type of nucleotitede sequences | Seuqnces deposited in GenBank | Genetic method | Reference |
|---|---|---|---|---|---|---|
| Pacific Ocean | surface | | 16S rRNA | Yes | PCR, DGGE | [46] |
| Saline lakes, coastal marine basin (Antarctica) | surface | | 16S rRNA | Yes | Cloning, RFLP | [47] |
| Freshwater lake (Keyna) | 0.10 | not known | 16S rRNA, *mcyE/ndaF* | Yes | PCR-DGGE | [48] |
| Saline lakes (USA) | 0.10 | 40 BP | *mcyA, mcyD,* 16S rRNA | Yes | PCR, qPCR | [49] |
| Freshwater lakes (France) | 0.25 | 90 BP | 16S rRNA-ITS, *mcyA*, PC-IGS | Yes | PCR, qPCR | [50] |
| Freshwater lake (France) | 0.40 | 100 BP | 16S rRNA, ITS 1 | Yes | PCR, qPCR | [51] |
| Freshwater Lagoon (Urugway) | 0.50 | 170 BP | 16S-23S - ITS, *sxtU* | Yes | Cloning, PCR, qPCR, DGGE | [52] |
| Freshwater lakes (Switzerland) | 1.00 | 200 BP | 16S rRNA, *mcyA* | Yes | PCR | [53] |
| Freshwater lakes (Norway) | 0.45 | 400 BP | *ociB* | No | PCR, qPCR | [54] |
| Freshwater lake (Canada) | 0.40 | 440 BP | 16S rRNA, *glnA* | No | PCR, qPCR | [55] |
| Saline lakes (Antarctica) | 2.45 | 3000 BP | 16S rRNA | Yes | PCR-DGGE | [56] |
| Saline lake (China, Asia) | 5.00 | 3000 BP | 23S rRNA | No | PCR, qPCR, DGGE | [57] |
| Baltic Sea (Europe) | 4.00 | >4000 BP | 16S rRNA, *nadF/mcyE,* PC-IGS | Yes | PCR | This work |
| Baltic Sea (Europe) | 5.50 | 8500-7400 BP | 16S rRNA | Yes | Cloning, T-RFLP | [28] |
| Saline lake (Antractica) | 1.50 | 10 000 BP | 16S rRNA | Yes | PCR, qPCR, DGGE | [58] |
| Saline lake (China) | 5.60 | 18 500 BP | 23S rRNA | Yes | PCR-DGGE | [59] |
| *Marine evaporites (Italy) | | 5.9-5.8 Ma | 16S rRNA | Yes | Cloning, qPCR | [27] |

**Table S5:** Modifications made to manufactures instructions during the isolation of DNA with two different kits.

| NucleoSpin® Soil | FastDNA™ Kit for Soil |
|---|---|
| The effect of both buffers (SL1 and SL2) provided with the kit was tested | Sample was homogenized by vortexing (2 × 1 min), instead of application of FastPrep Instrument (step 4) |
| The enhancer SX provided with the kit was not used (step 2 of the instruction) | DNA was eluted from NucleoSpin® Column with 50 μl of DES |
| DNA was eluted from NucleoSpin® Column with 50 μl of DNase/Pyrogen-Free Water (DES) instead of suggested buffer SE | |
